# High‐Valence‐Manganese Driven Strong Anchoring of Iridium Species for Robust Acidic Water Oxidation

**DOI:** 10.1002/advs.202205920

**Published:** 2023-01-22

**Authors:** Yuxiao Weng, Keyu Wang, Shiyi Li, Yixing Wang, Linfeng Lei, Linzhou Zhuang, Zhi Xu

**Affiliations:** ^1^ State Key Laboratory of Chemical Engineering School of Chemical Engineering East China University of Science and Technology Shanghai 200237 China

**Keywords:** acidic oxygen evolution reaction, anchor sites, high‐valence manganese, Mn–O–Ir coordination

## Abstract

Designing an efficient and durable electrocatalyst for the sluggish anodic oxygen evolution reaction (OER) has been the primary goal of using proton exchange membrane electrolyzer owing to the highly acidic and oxidative environment at the anode. In this work, it is reported that high‐valence manganese drives the strong anchoring of the Ir species on the manganese dioxide (MnO_2_) matrix via the formation of an Mn–O–Ir coordination structure through a hydrothermal‐redox reaction. The iridium (Ir)‐atom‐array array is firmly anchored on the Mn–O–Ir coordination structure, endowing the catalyst with excellent OER activity and stability in an acidic environment. Ir‐MnO_2_(160)‐CC shows an ultralow overpotential of 181 mV at *j* = 10 mA cm^−2^ and maintains long‐term stability of 180 h in acidic media with negligible decay, superior to most reported electrocatalysts. In contrast, when reacting with low‐valence MnO_2_, Ir species tend to aggregate into IrO*_x_* nanoparticles, leading to poor OER stability. Density functional theory (DFT) calculations further reveal that the formation of the Mn–O–Ir coordination structure can optimize the adsorption strength of *OOH intermediates, thus boosting the acidic OER activity and stability.

## Graphical abstract

High‐valence‐manganese can drive the strong anchoring of Ir species on the manganese dioxide (MnO_2_) matrix via forming the Mn–O–Ir coordination structure through a hydrothermal‐redox reaction, endowing the catalyst with excellent OER activity and stability in acidic environments. The as‐prepared Ir‐MnO_2_(160)‐CC exhibits an ultralow overpotential of 181 mV at *j*=10 mA cm^−2^ and operated stably for 180 h with negligible decay.

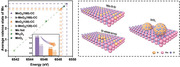

## 1 Introduction

Among the various water electrolysis techniques, the proton exchange membrane water electrolyzer (PEMWE) exhibits the highest purity and efficiency of H_2_ production and the best high‐pressure tolerance.^[^ ^1^, ^2^, ^3^ ^]^ However, the sluggish kinetics of the oxygen evolution reaction (OER) at the anode, which results from its complicated four‐electron transfer, becomes a major bottleneck for improving the energy conversion efficiency of water electrolyzers.^[^ ^4^, ^5^, ^6^, ^7^, ^8^ ^]^ Meanwhile, PEMWE usually performs in a harsh acidic environment at a high anode potential, leading to a significant stability challenge for acidic OER (AOER) catalysts.^[^ ^3^, ^9^ ^]^ Most transition metal catalysts with excellent OER activity in alkaline electrolytes, such as Fe‐ and Ni‐based catalysts, are vulnerable to dissolution and deactivation in an acidic medium.^[^ ^10^, ^11^, ^12^, ^13^ ^]^ Noble‐metal‐based OER electrocatalysts, such as Ir, Ru, and their oxides, exhibit relatively better tolerance to acidic media. However, RuO_2_ can still be oxidized to RuO_4_ under a high anodic potential and is thus considered unstable in acids.^[^ ^14^, ^15^, ^16^ ^]^ Instead, Ir‐based materials show relatively high stability, but the simultaneous achievement of excellent catalytic activity and robust durability remains a major challenge.

Accordingly, many studies have focused on developing Ir‐based catalysts with balanced activity and stability, such as in situ reconstructions,^[^ ^17^ ^]^ heterojunction structures,^[^ ^18^ ^]^ and optimization of electronic structures.^[^ ^19^ ^]^ The construction of strong interactions between the Ir species and the support is considered an effective strategy. The strong interaction helps enhance the dispersion of Ir sites, thus preventing the aggregation of the catalytic material on the support, while the unique electronic structure promotes the rapid transfer of electrons.^[^ ^14^ ^]^ A promising strategy for synthesizing AOER catalysts with strong interactions is to achieve activity‐stability optimization by loading Ir species on acid‐resistance supports.^[^ ^4^, ^20^, ^21^ ^]^ Generally, acid‐resistant oxides based on elements such as Ti,^[^ ^22^ ^]^ W,^[^ ^19^ ^]^ and Mo^[^ ^23^ ^]^ cannot withstand highly oxidizing and harsh acidic conditions in long‐term durability tests. Kim et al.^[^ ^22^ ^]^ prepared TiO_2_‐MoO*_x_* as the support for Ir nanoparticles, maintaining a 10 h stability at 10 mA cm^−2^. Liu et al.^[^ ^23^ ^]^ prepared Ir (Rh, Au, Ru)‐MoO_3_ embedded in graphitic carbon layers, demonstrating an ultralow overpotential of 156 mV but 40 h stability at 10 mA cm^−2^. However, Mn could exist in a variety of valence states and crystalline phases, while MnO_2_ can operate stably at pH 0–14 from 1.3 to 1.7 V versus RHE, a highly suitable and promising material for AOER catalysts.^[^ ^11^, ^24^, ^25^ ^]^ Generally, the OER activities of MnO_2_ strongly depend on the crystallographic structures, following with an order of *α*‐MnO_2_ > amorphous > *β*‐MnO_2_ > *γ*‐MnO_2_ > *δ*‐MnO_2_.^[^ ^26^, ^27^ ^]^ Inspired by the above studies, Mn, with high acid resistance and redox activity, is suggested as a promising alternative component to support Ir sites for enhancing the activity and stability toward the AOER.

Herein, we used *α*‐MnO_2_ as the substrate and applied the redox reaction to form an Mn–O–Ir coordination structure, which can further act as the anchoring site of the Ir atom array. The valence of Mn was manipulated by introducing potassium permanganate as an oxidizing agent and controlling the temperature. Through comprehensive experimental characterization, the high‐valence Mn species were verified to be crucial in driving the formation of Mn–O–Ir structures, further leading to the strong interaction between the Ir atom array and the *α*‐MnO_2_ support, endowing the catalysts with promising catalytic activity and stability. The obtained Ir‐MnO_2_(160)‐CC showed excellent OER activity in 0.5 m H_2_SO_4_, with an overpotential (*η*) of only 181 mV versus RHE to achieve a current density of 10 mA cm^−2^, which is better than that of other reported AOER electrocatalysts. Moreover, Ir‐MnO_2_(160)‐CC also exhibited robust long‐term stability in 0.5 m H_2_SO_4_, with a negligible potential increase after 180 h of operation at *j* = 20 mA cm^−2^. In contrast, when reacting with low‐valence Mn species, Ir species tended to aggregate into IrO*_x_* nanoparticles rather than forming Mn–O–Ir coordination, leading to poor OER activity and stability. Density functional theory (DFT) calculations further revealed that the valence state of Mn indeed decreased after doping with Ir atoms, and the formed Mn–O–Ir coordination could optimize the adsorption strength of *OOH intermediates, leading to their excellent activity.

## 2 Results and Discussion

### 2.1 Morphological and Structural Characterization

**Figure 1:**
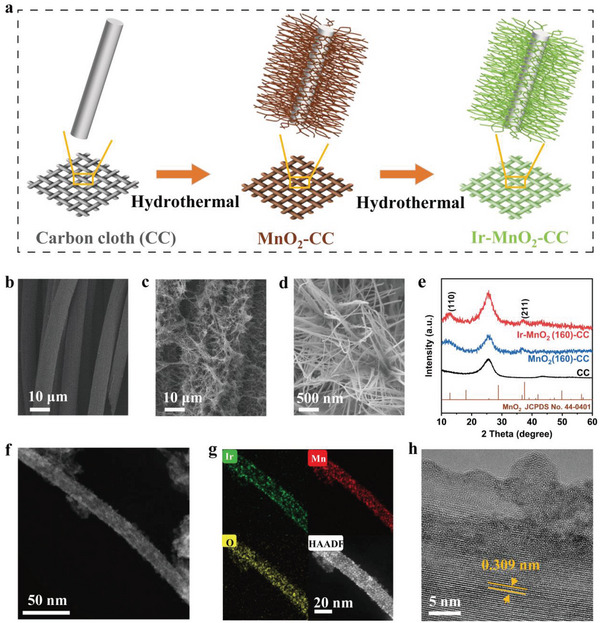
The synthesis route and morphological characterization. a) The synthesis procedure for the electrocatalysts. b) SEM of pure carbon cloth. c,d) SEM of MnO_2_(160)‐CC. e) XRD patterns. f) HAADF STEM of Ir‐MnO_2_(160)‐CC. g) corresponding mapping images. h) HRTEM image.

As shown in **Figure** **1**a, Ir species‐anchored MnO_2_ on the carbon cloth catalysts (Ir‐MnO_2_(160)‐CC) were synthesized using a hydrothermal strategy. Based on the reported methods,^[^ ^28^, ^29^ ^]^ MnO_2_(160)‐CC was prepared through the redox reaction of carbon cloth (denoted as CC) in the presence of aqueous KMnO_4_ at 160 °C. CC color changed significantly from black to brown (Figure S1, Supporting Information). The obtained MnO_2_(160)‐CC was then immersed in an aqueous solution of K_2_IrCl_6_ ·*x*H_2_O for another hydrothermal treatment to load the Ir species. The scanning electron microscopy (SEM) image (Figure 1b) showed that pure CC had a 3D porous architecture with a smooth surface and distinct boundaries between the fibers. After reacting with KMnO_4_, the surface of the carbon fiber was thickly coated with large amounts of ultrafine nanowires (Figure 1c,d). The morphology of the nanowires remained unchanged after reaction with K_2_IrCl_6_ ·*x*H_2_O (Figure S2, Supporting Information). X‐ray diffraction (XRD) patterns of MnO_2_(160)‐CC and Ir‐MnO_2_(160)‐CC (Figure 1e) showed peaks at 12.78° and 37.52°, corresponding to the (110) and (211) planes of *α*‐MnO_2_ (JCPDS No. 44–0141), respectively.^[^ ^30^, ^31^ ^]^ Meanwhile, owing to the high crystallinity of carbon, the peak at 25.5° ascribed to CC was much stronger than that of MnO_2_(160). Moreover, no Ir‐related peaks were detected, indicating high dispersion of the Ir species. The high‐angle annular dark field (HAADF) STEM images (Figure 1f) further confirmed the ultrafine nanowire structure of MnO_2_(160) with a diameter of ≈6–8 nm (Figure S3, Supporting Information), consistent with recent reports.^[^ ^25^, ^32^ ^]^ As shown in Figure 1g, the corresponding elemental mapping revealed the homogeneous distribution of Ir, Mn, and O elements, confirming the successful synthesis of Ir‐MnO_2_(160)‐CC and the uniform distribution of Ir species on the MnO_2_ nanowires. From the high‐resolution transmission electron microscopy (HRTEM) images (Figure 1h; Figure S4, Supporting Information), ordered lattice fringes with an interplanar spacing of 0.309 nm were observed, corresponding to the MnO_2_ (310) plane. The bright protrusions in Figure 1h were attributed to Ir species. Furthermore, the inductively coupled plasma (ICP) tests confirmed the content of Ir and Mn in the obtained Ir‐MnO_2_(160)‐CC to be 2.7 and 7.7 wt%, respectively (Table S1, Supporting Information). Thus, the atomic ratio of Ir/Mn was calculated to be 1:10, consistent with the theoretical feed metal mole ratio.

**Figure 2:**
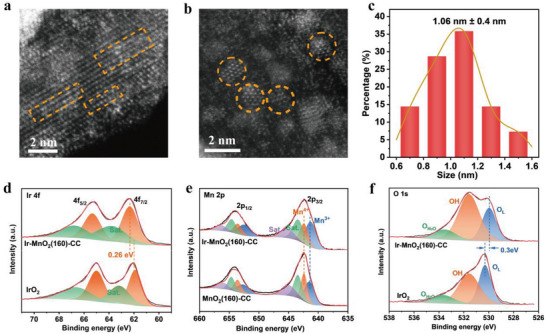
The morphological and structure characterization. a,b) Aberration‐corrected HAADF‐STEM image of Ir‐MnO_2_(160)‐CC. c) The size distribution of Ir‐atom‐array. d) The high‐resolution Ir 4f XPS spectra. e) The high‐resolution Mn 2p XPS spectra. f) The high‐resolution O 1s XPS spectra.

Aberration‐corrected HAADF‐STEM was used to image the atomic structure of the Ir‐MnO_2_(160)‐CC. As in the HAADF‐STEM images, the signal intensity was proportional to the square of the atomic number; Ir atoms were brighter than Mn atoms, while lighter O atoms were not visible. As shown in **Figure** **2**a,b and Figure S5 (Supporting Information), abundant Ir atom arrays consisting of 8 to 10 Ir atoms were found to form on the (310) crystal plane of MnO_2_, giving a size distribution of 1.06 ±0.40 nm (Figure 2c). The elemental mapping data from the SEM and XPS survey spectra (Figures S6 and S7, Supporting Information) further confirmed the homogeneous distribution of C, Ir, Mn, and O atoms in the sample, indicating the successful synthesis of Ir‐MnO_2_(160)‐CC. The Ir 4f XPS spectra (Figure 2d) of Ir‐MnO_2_(160)‐CC and commercial IrO_2_ particles showed two peaks corresponding to the 4f_7/2_ and 4f_5/2_ states separated by 3.2 eV, decoupled into four sets of doublet peaks. For Ir‐MnO_2_(160)‐CC, the peaks at 62.13 eV/65.33 eV were identified as Ir^4+^ species,^[^ ^33^, ^34^ ^]^ which were 0.26 eV higher than those of commercial IrO_2_ catalyst (61.87 eV/65.07 eV), indicating that the confined Ir species in MnO_2_ had a higher oxidation state than that in IrO_2_ particles,^[^ ^35^, ^36^ ^]^ possibly attributed to the formation of an Mn–O–Ir coordination structure.^[^ ^37^ ^]^ Meanwhile, for the Mn 2p XPS spectra of Ir‐MnO_2_(160)‐CC and MnO_2_(160)‐CC (Figure 2e), the fitted peaks at 641.2 eV/652.4 eV and 642.3 eV/ 653.5 eV could be ascribed to Mn^3+^ and Mn^4+^. The atomic ratio of Mn^4+^/Mn^3+^ decreased from 0.8:1 to 0.6:1, indicating a decrease in the oxidation state of Mn after loading the Ir species. Combined with the increased oxidation state of Ir in Ir‐MnO_2_(160)‐CC, it was proposed that a redox reaction should occur between Ir and MnO_2_. The O 1s spectra (Figure 2f) confirmed that the chemical states of O in Ir‐MnO_2_(160)‐CC and IrO_2_ were apparently different because the lattice oxygen (O_L_) of crystalline IrO_2_ was located at 530.2 eV, while the O_L_ for Ir‐MnO_2_(160)‐CC appeared at the shoulder (529.9 eV). The binding energy of the main peak in Ir‐MnO_2_(160)‐CC was positively shifted, which was usually assigned to the hydroxyl groups.^[^ ^38^ ^]^

### 2.2 Acidic OER Performances

**Figure 3:**
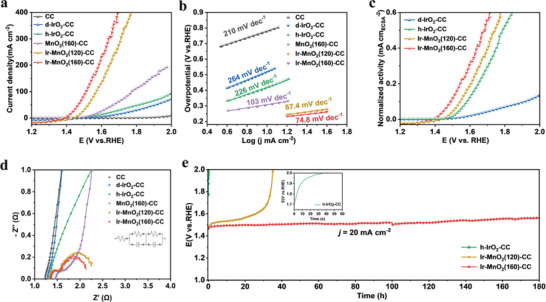
The OER performance for the prepared catalysts. a) CV curves of all catalysts in 0.5 m H_2_SO_4_ at a scan rate of 5 mV s^−1^. b) Tafel plots derived from a). c) Normalized activity. d) Nyquist plots for the catalysts in 0.5 m H_2_SO_4_ at ≈1.4 V versus RHE. e) Chronopotentiometric curves at 20 mA cm^−2^.

The AOER performance of Ir‐MnO_2_(160)‐CC was tested in 0.5 m H_2_SO_4_ at room temperature using a typical three‐electrode system. For comparison, Ir‐MnO_2_(120)‐CC (prepared following the same procedure, but the hydrothermal reaction temperature for preparing MnO_2_ was 120 °C), MnO_2_(160)‐CC, homemade IrO_2_‐CC (h‐IrO_2_‐CC) obtained by hydrothermal synthesis, commercial IrO_2_ dropped onto a carbon cloth (d‐IrO_2_‐CC), and pure CC (details provided in the Experimental Section) were used as contrast samples. The cyclic voltammetry (CV) curves of the catalysts before and after 0.9*iR correction are provided in **Figure** **3**a and Figure S8 (Supporting Information). Figure 3a showed that Ir‐MnO_2_(160)‐CC exhibited the best AOER activity with the lowest overpotential (*η*_10_) of 181 mV, clearly lower than those of Ir‐MnO_2_(120)‐CC (236 mV), MnO_2_(160)‐CC (417 mV), h‐IrO*_x_* (307 mV), d‐IrO*_x_* (336 mV), and CC (780 mV), and even much smaller than those of recently reported catalysts (Table S2, Supporting Information). In addition, as shown in Figure 3b, the Tafel slope of Ir‐MnO_2_(160)‐CC (74.8 mV dec^−1^) was much lower compared to Ir‐MnO_2_(120)‐CC (87.4 mV dec^−1^), MnO_2_(160)‐CC (103 mV dec^−1^), h‐IrO*_x_* (226 mV dec^−1^), d‐IrO*_x_* (264 mV dec^−1^), and CC (210 mV dec^−1^), indicative of its fastest charge transfer. The superior reaction kinetics were further explored through electrochemically active surface areas (ECSA) by obtaining the double‐layer capacitance (*C*_dl_) of the samples from the cyclic voltammograms (CVs) with varied scan rates (Figures S9 and S10 and Table S3, Supporting Information). When normalizing the OER activity of the catalysts to the ECSA, the specific activity at 1.63 V versus RHE of Ir‐MnO_2_(160)‐CC (0.38 mA cm_ECSA_^−2^) was found to be 1.46 times, 2.23 times and 14.07 times greater than those of Ir‐MnO_2_(120)‐CC (0.26 mA cm_ECSA_^−2^), h‐IrO*_x_* (0.17 mA cm_ECSA_^−2^), and d‐IrO*_x_* (0.027 mA cm_ECSA_^−2^) (Figure 3c). After loading Ir species, the mass activity calculated at 1.53 V versus RHE for Ir‐MnO_2_(160)‐CC was approximately 343.4 A g_Ir_^−1^cm^−2^, 50.5 times, and 68.7 times greater than that of h‐IrO_2_‐CC (6.8 A g_Ir_^−1^ cm^−2^), and d‐IrO_2_‐CC (5.0 A g_Ir_^−1^ cm^−2^) (Figure S11, Supporting Information). Ir‐MnO_2_(120)‐CC showed better mass activity (437.8 A mg_Ir_^−1^ cm^−2^ at 1.53 V vs RHE); however, considering the same feeding amount of Ir precursor during the preparation process, its remarkably lower Ir mass fraction (0.9 wt%) indicated a huge waste of the precious metal (Figure S12 and Table S4, Supporting Information). The electrochemical impedance spectroscopy (EIS) analysis showed that the electronic conductivity of Ir‐MnO_2_(160)‐CC could be enhanced with low solution resistance (*R*_s_) of 1.3 Ω and low charge‐transfer resistance (*R*_ct_) of 0.85 Ω (Figure 3d; Figure S13 and Table S4, Supporting Information). The TOF values have been calculated for all the catalysts.^[^ ^4^, ^39^ ^]^ The TOF of Ir‐MnO_2_(160)‐CC reached up to 0.321 s^−1^ at the overpotential of 370 mV (vs RHE), remarkably higher than that of h‐IrO_2_‐CC (0.01 s^−1^) and d‐IrO_2_‐CC (0.005 s^−1^). The above results indicate that Ir‐MnO_2_(160)‐CC could serve as a promising OER electrocatalyst for water splitting in acidic media.

In addition to catalytic activity, stability is another important parameter for evaluating whether an AOER catalyst is promising. As shown in Figure 3e, Ir‐MnO_2_(160)‐CC showed negligible potential change to achieve *j =* 20 mA cm^−2^ within 180 h of operation in 0.5 m H_2_SO_4_, while h‐IrO_2_‐CC decayed quickly within 1 min under the same acidic environment, demonstrating the importance of anchoring Ir species on acidic‐resistant support. In proton exchange membrane electrolyzer, the Ir‐MnO_2_(160)‐CC and Pt/C were applied as the anode and cathode, respectively. Ir‐MnO_2_(160)‐CC // Pt/C could achieve the current density of 100 mA cm^−2^ at the small cell voltage of 1.65 V, and show negligible decay in 70 h, indicative of its potential for practical hydrogen production via PEMWE (Figure S14, Supporting Information). Interestingly, Ir‐MnO_2_(120)‐CC could only operate stably for less than 10 h at *j =* 20 mA cm^−2^, after which the required voltage increased rapidly. As the preparation conditions of Ir‐MnO_2_(160)‐CC and Ir‐MnO_2_(120)‐CC were nearly the same, except for the hydrothermal synthesis temperature of MnO_2_, the intrinsic properties of MnO_2_ may significantly affect the loading process of Ir species, resulting in significantly different activities and stabilities of the obtained Ir‐MnO_2_‐CC samples.

### 2.3 Mechanism Study

**Figure 4:**
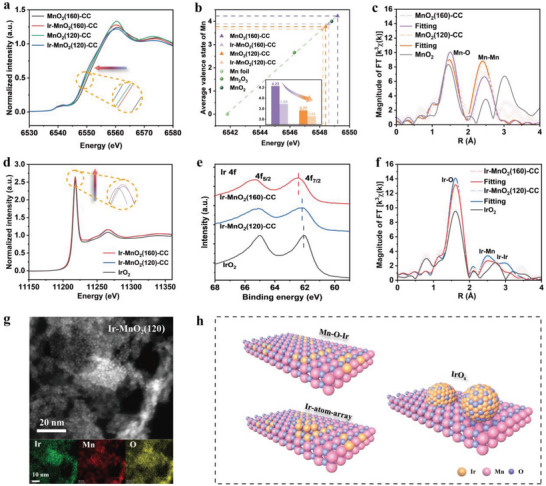
Mechanistic study of catalytic active sites. a) Normalized Mn *K*‐edge XANES spectra. b) Average valence state of Mn. c) The *k* ^3^‐FFT EXAFS spectra of the Mn *R*‐space and corresponding fitting curves. d) Normalized Ir L‐edge XANES spectra. e) The high‐resolution Ir 4f XPS spectra. f) The *k* ^3^‐FFT EXAFS spectra of the Ir *R*‐space and corresponding fitting curves. g) TEM image and corresponding mapping images of Ir‐MnO_2_(120)‐CC. h) Schematic diagram of the three existing forms of Ir species.

X‐ray absorption spectroscopy (XAS) was used to investigate the valence states and fine structure of the Ir‐MnO_2_‐CC samples to reveal the root cause of their distinct stability. As shown in Figure S15 in the Supporting Information, the crystallinities of MnO_2_(160)‐CC and MnO_2_ (120)‐CC were almost the same. In contrast, from the normalized X‐ray absorption near‐edge structure (XANES) spectra of the Mn *K*‐edge (**Figure** **4**a), the valence state of Mn in MnO_2_(160)‐CC was much higher than that in MnO_2_(120)‐CC, possibly contributing to the distinct differences in their stabilities. Therefore, the linear relationship between the Mn valence state and energy was fitted using the XANES data of the Mn foil, Mn_2_O_3_, and MnO_2_ standard samples (Figure 4b). The valence state of Mn in MnO_2_(160)‐CC reached 4.23, clearly higher than that in MnO_2_(120)‐CC (3.77). Meanwhile, in the extended X‐ray absorption fine structure (EXAFS) data (Figure 4c; Figure S16, Supporting Information), the peak of the Mn–O bond in MnO_2_(160)‐CC was significantly higher, further confirming a larger number of O coordination compared with MnO_2_(120)‐CC (Table S5, Supporting Information). After the redox reaction, the Mn valence state in Ir‐MnO_2_(160)‐CC would decrease to 3.89, with a valence decrease of 0.34 (Inset Figure 4b), while the valence decrease for Ir‐MnO_2_(120)‐CC would be merely 0.11. In return, the normalized Ir *L*‐edge XANES spectra (Figure 4d) prove that Ir‐MnO_2_(160)‐CC possessed a higher valence state of Ir than Ir‐MnO_2_(120)‐CC and IrO_2_. These results demonstrated that high‐valence Mn could promote the redox reaction of Mn ions with IrCl_6_^2−^ to form an Mn–O–Ir coordination structure, contributing more anchoring sites for the extra Ir ions to form an Ir atom array. A lower hydrothermal temperature leaded to an obviously lower valence state of Mn and a lower driving force for the redox reaction to occur. As Mn and Ir atoms exhibited a significant difference in electronegativity (1.881 for Ir^4+^ and 1.912 for Mn^4+^),^[^ ^37^, ^40^ ^]^ the formation of the Mn–O–Ir coordination structure should redistribute the electron charge, leading to a positive shift of the Ir peak in the XPS Ir 4f spectrum compared with IrO_2_ (Figure 4e). In addition, in the *k* ^3^‐FFT EXAFS spectra of the Ir *R*‐space (Figure 4f), Ir–Mn and Ir–O scattering paths were observed in Ir‐MnO_2_‐CC, demonstrating that the presence of Mn–O–Ir. The Ir–Ir distance was closer to the Ir–Ir distance obtained from IrO_2_ CIF and the Ir–Ir scattering path in ≈2.8 Å was consistent with that in *k* ^3^‐FFT Ir *R*‐space EXAFS spectra of pure IrO_2_, thus we can confirm the existence of Ir atom array. The *k*‐space *χ*(*k*) curves of EXAFS oscillation functions are shown in the Figure S17 (Supporting Information). For Ir‐MnO_2_(120)‐CC with insufficient Mn–O–Ir anchoring sites, the Ir species preferred to form larger particles directly anchored on MnO_2_. The HRTEM and SEM images clearly demonstrated the presence of these large nanoparticles (with a diameter of ≈20 nm) in Ir‐MnO_2_(120)‐CC (Figure 4g; Figure S18, Supporting Information). According to the above characterization results, the Ir species should exist in the following four forms (Figure 4h): including Mn–O–Ir coordinations (the ideal existence form, confirmed by XAS analysis, Figure 4f), Ir atom arrays (confirmed by AC‐TEM analysis, Figure 2a,b and Figure S5, Supporting Information), and IrO*_x_* nanoparticles anchored on MnO_2_ (confirmed by HRTEM and SEM, Figure 4g and Figure S18, Supporting Information). Considering the major difference between Ir‐MnO_2_(160)‐CC and Ir‐MnO_2_(120)‐CC is whether the Mn–O–Ir coordination structure can be constructed through a redox reaction, the strong interaction formed between the Ir‐atom‐array and MnO_2_ connected by the Mn–O–Ir structure should play a crucial role in endowing Ir‐MnO_2_(160)‐CC with clearly superior OER stability. Additionally, Ir‐MnO_2_(140)‐CC and Ir‐MnO_2_(180)‐CC were synthesized using a similar method, and their OER performances were tested (Figures S19 and S20, Supporting Information). The performance and stability of Ir‐MnO_2_(140)‐CC were slightly inferior to those of Ir‐MnO_2_(160)‐CC, but the stability of Ir‐MnO_2_(180)‐CC dropped sharply in comparison to Ir‐MnO_2_(160)‐CC because of the serious rupture of MnO_2_ at overhigh hydrothermal temperatures. Without carbon cloth, the Mn–O–Ir coordination may not be formed following the same procedure (Figure S21, Supporting Information).

**Figure 5:**
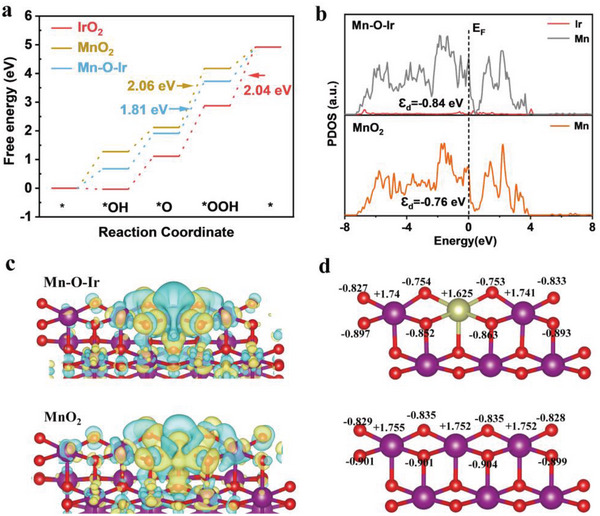
DFT calculations. a) Gibbs free‐energy diagram for IrO_2,_ MnO_2_ and Ir‐MnO_2_. b) The calculated pDOS curves for MnO_2_ and Ir‐MnO_2_. c). The charge density difference plots for Ir‐MnO_2_ and MnO_2_. Yellow and blue contours represent electron accumulation and depletion, respectively. d) The differential charge density at Mn, O and Ir sites.

DFT calculations were performed to study the Gibbs free energies of the OER elementary steps and the overpotential for a confined Ir atom in the MnO_2_ crystalline matrix to better understand the underlying mechanism of the catalytic performance improvement for Ir‐MnO_2_‐CC in acidic media; the calculation methods are described in the Experimental Section. After a series of optimizations, the (310) plane of *α*‐MnO_2_ was chosen as the surface model for subsequent calculations. With one Mn atom in the MnO_2_ unit cell substituted by one Ir atom, we obtained the structure of Mn–O–Ir. An IrO_2_ (111) model was also constructed for comparison (Figure S22, Supporting Information). Four consecutive elementary electron steps occurred at Ir sites in the AOER process, which followed the conventional adsorbate evolution mechanism (AEM) pathway (Figure S23, Supporting Information).^[^ ^36^, ^41^, ^42^ ^]^ Gibbs free energy (Δ*G*) plots of the four elementary steps are shown in **Figure** **5**a. For the MnO_2_ and Ir‐MnO_2_, the formation of Ir‐OOH* intermediate from Ir‐O* was the OER rate‐determining step (RDS), and their free energy barriers were 2.06 and 1.81 eV, respectively. The RDS of IrO_2_ was the formation of O_2_ from OOH*, which needed 2.04 eV to overcome the energy barrier. The clearly lower energy barrier of Ir‐MnO_2_ verified that the Mn–O–Ir structure can optimize the intermediate adsorption strength and boost the OER activity. Moreover, to better understand the discrepancy in the oxygen adsorption energy, the partial density of states (PDOS) of the Mn 3d and Ir 5d orbitals in the models of MnO_2_, IrO_2_, and Ir‐MnO_2_ were calculated (Figure 5b; Figure S24, Supporting Information), which were directly associated with the OER intrinsic activity.^[^ ^43^, ^44^ ^]^ Compared to the d‐band center (*Ɛ*_d_) of IrO_2_ (− 2.41 eV) and MnO_2_ (− 0.76 eV), the *Ɛ*_d_ for Ir‐MnO_2_ had a favorable value of − 0.84 eV, further confirming a charge redistribution at Ir active site and a optimize of the adsorption energy of Ir active site to oxygen intermediates.^[^ ^44^ ^]^ From the charge density difference plots of Ir‐MnO_2_ and MnO_2_ in Figure 5c, the electron cloud density around the Ir site decreased compared to that around the Mn site, coinciding with the data mentioned above for the electronegativity of Ir^4+^ and Mn^4+^. After Ir doping, the differential charge density at the Mn site decreased from +1.753 |e| to +1.74 |e|, and the Ir site in MnO_2_ had +1.625 |e|, higher than the +1.556 |e| of Ir sites in IrO_2_ (Figure 5d; Figure S25, Supporting Information). Moreover, in Figure S26 (Supporting Information), another configuration for configuration of Ir‐atom‐array model was calculated, in which 4 Mn atoms were substituted by 4 Ir atoms in the MnO_2_, and it was found that the OER rate‐determining step was the deprotonation of Ir–OH* to form Ir–O* intermediate with a higher free energy barrier (1.92 eV). Combined with the results in Figure 5a, it could be concluded that the most active site was Mn–O–Ir, followed by the Ir–O–Ir structure in the form of Mn–O–(Ir‐atom‐array). They both possessed higher OER activity than IrO_2_ and MnO_2_. The results calculated by DFT were consistent with the experimental and characterization results.

The spent Ir‐MnO_2_(160)‐CC was characterized using TEM, XRD, AC‐TEM, and XPS after a long‐term stability test. Clearly, the nanowire retained its morphological integrity after 180 h of operation, and all the elements could maintain a uniform distribution (Figure S27, Supporting Information), verifying the excellent stability of Ir‐MnO_2_(160)‐CC toward the OER in acidic and highly oxidizing conditions. The HRTEM image of the spent Ir‐MnO_2_(160)‐CC suggested the integrity of the crystal lattice (Figure S28a, Supporting Information). Meanwhile, the crystal structure of spent Ir‐MnO_2_(160)‐CC remained unchanged, as determined by XRD (Figure S28b, Supporting Information). Furthermore, the AC‐TEM image (Figure S29, Supporting Information) showed the Ir‐atom‐array with a slightly larger average particle size (1.45 ±0.25 nm) on the surface of the MnO_2_ nanowire, illustrating a strong interaction between Ir species and MnO_2_ nanowire, with no local aggregation during the long period of the OER process. The robust durability of Ir‐MnO_2_(160)‐CC was confirmed by XPS (Figure S30, Supporting Information). The XPS survey spectra of the spent catalyst were similar to those of the original catalyst. The ratio of Ir/Mn in spent Ir‐MnO_2_(160)‐CC was close to that of the fresh electrocatalyst, while the peaks of Ir 4f and Mn 2p for spent Ir‐MnO_2_(160)‐CC experienced slightly positive shifts after the durability test. Moreover, after the catalyst stabilized for 180 h, a reduction in the concentration of lattice oxygen (6.62%) was observed because MnO_2_ experienced slight damage under harsh acidic conditions and high anodic potential. The above characterization results confirmed that the strong interaction between the Ir species and MnO_2_ endows Ir‐MnO_2_(160)‐CC with excellent stability toward the acidic OER.

## 3 Conclusions

In conclusion, we constructed a Mn–O–Ir coordination structure on high‐valence‐state MnO_2_ nanowires via a redox reaction to form an Ir atom array. Charge transfer between the Mn and O–Ir coordination structures could regulate the unsaturated coordination environment of Ir. Ir‐MnO_2_(160)‐CC exhibits excellent OER performance in 0.5 m H_2_SO_4_ and promising long‐term stability for maintaining 180 h at *j* = 20 mA cm^−2^. In sharp contrast, Ir‐MnO_2_(120)‐CC, which has a lower valence of Mn, is more inclined to form IrO*_x_* particles while exhibiting inferior OER activity and stability (<10 h) in 0.5 m H_2_SO_4_. Detailed characterizations of the spent catalyst also demonstrate the promising stability of the confined Ir atom array on the MnO_2_ nanowires. DFT simulations indicate that the adsorption strength of the *OOH intermediates could be optimized by anchoring atomic Ir on MnO_2_. This strategy of introducing suitable acid‐resistant substrates, such as MnO_2_, to confine Ir species on carbon cloth might apply to other noble metal sites for developing various electrocatalytic reactions.

## 4 Experimental Section

### Materials and Chemicals

Dipotassium hexachloroiridate (K_2_IrCl_6_·*x*H_2_O), iridium oxide (IrO_2_), potassium permanganate (KMnO_4_), and H_2_SO_4_ (98 wt.∖%) were purchased from Aladdin. The WOS1009 carbon cloth was purchased from Shanghai Hesen Co., Ltd, whose main parameters include: thickness (330 mm), basic weight (120 g cm^−2^), air permeability (<10 s), through‐plane resistance (<5 mΩ cm^−2^), tensile strength (MD, 10 N cm^−1^), and tensile strength (XD, 5 N cm^−1^). All the chemicals and reagents were used as received without further purification.

### Pretreatment of CC

The carbon cloth was immersed in an acid solution containing 20 mL 10 wt% HNO_3_ and 60 mL 10 wt% H_2_SO_4_ for 12 h at 80 °C to improve its hydrophilicity.

### Synthesis of MnO_2_‐CC

50 mg KMnO_4_ was added to deionized water (60 mL) and stirred for 30 min to generate a uniform purple solution. The treated CC was then placed in the obtained solution, and the mixed system was sealed and maintained at 160 °C for 12 h in an autoclave. For MnO_2_(120)‐CC, the synthesis processes were the same except that the hydrothermal temperature for obtaining MnO_2_‐CC was 120 °C.

### Synthesis of Ir‐MnO_2_(160)‐CC

The obtained clean MnO_2_‐CC electrode (1×3 cm) was immersed in a K_2_IrCl_6_.*x*H_2_O water solution (5 mg, 20 ml) at 110 °C for 6 h. For Ir‐MnO_2_(120)‐CC, the synthesis processes were the same, except that the applied substrate was MnO_2_(120)‐CC.

### Synthesis of h‐IrO_2_‐CC

The clean pretreated CC electrode (1×3 cm) was directly immersed in a homogeneous K_2_IrCl_6_·*x*H_2_O solution (5 mg, 20 mL). The system was sealed at 110 °C for 6 h.

### Synthesis of d‐IrO_2_‐CC

The IrO_2_ (10 mg) was dispersed in Nafion117 (5%, 100 µL) and isopropyl alcohol (900 µL) solution by ultrasonication for 30 min to obtain a homogenous catalyst ink. The above suspension (150 µL) was then dropped onto the CC electrode (1×1 cm^2^). The catalyst load was 1.28 mg_Ir_ cm^−2^ by weighing the quality before and after dripping and calculation. All obtained catalysts were washed with water and ethanol and dried in an oven at 60 °C before OER testing.

### Electrochemical Test

Catalyst performance was evaluated in a three‐electrode cell with a 0.5 m H_2_SO_4_ electrolyte on CHI 660E. A piece of the 1×1 cm^2^ catalyst was used directly as the working electrode. A graphite electrode and Ag/AgCl electrode were employed as the counter and reference electrodes, respectively. All potentials were calibrated with *iR* compensation and referenced to the reversible hydrogen electrode (*E*_RHE_ = 0.0591 pH + *E*(Ag/AgCl) + 0.2 − 0.9*iR). Cyclic voltammetry was conducted at a scan rate of 10 mV s^−1^. EIS was conducted in the frequency range of 10 kHz to 0.1 Hz at an overpotential of 100 mV. A specific capacitance of 1 cm^2^ flat surface area generally corresponds to a specific capacitance of 60 µF. Therefore, ECSA can be obtained from the following Equation: *R*_f_ = *C*_dl_/60 µF cm^−2^. *C*_dl_ was measured using the CV method in the non‐faradaic current region (0.85–0.95 V vs RHE) at scan rates of 10, 12, 14, 16, 18, and 20 mV s^−1^. Chronopotentiometry tests were performed at 20 mA cm^−2^ for 180 h to assess the long‐term water‐splitting stability of the catalysts. TOF values could be calculated by using following Equation (1):

(1) $${\mathrm{TOF}}_{\mathrm{OER}}=\left(i\times a\right)/\left(4\times n\times F\right)$$

where *i* represents the tested current density at given overpotential, *a* is the surfaces area of the working electrode (1 cm^2^), *n* is the mole number of active components on the working electrode, *F* is the Faraday constant (96 485 C mol^−1^).

### Characterization

X‐ray diffraction (XRD) patterns (2*θ*, 10°–80°) were measured using a Bruker D8‐Advanced X‐ray diffractometer. Scanning electron microscopy (SEM) was performed using a ZEISS Gemini 300 instrument (200 kV operating voltage). Transmission electron microscopy (TEM) and elemental mapping images were obtained using an HRTEM JEOL 2100F microscope. Aberration‐corrected HAADF‐STEM was operated on a Thermo Fisher Themis Z transmission electron microscope equipped with two aberration correctors. X‐ray photoelectron spectra were obtained using a Thermo Scientific K‐Alpha X‐ray photoelectron spectrometer (XPS) incorporating monochromatic Al K*α* (1486.6 eV) radiation at 72 W (12 kV, 6 mA). The binding energies were calibrated using the C 1s peak of adventitious carbon (284.8 eV) as the reference. The metal content was determined by inductively coupled plasma (ICP) mass spectroscopy using an Agilent 7800 ICP‐MS instrument.

### Computational Section

Density functional theory calculations were performed using the Vienna ab initio simulation package (VASP) program with the projector augmented wave (PAW) method, with the kinetic energy cutoff set to 450 eV. The convergence criterion for electronic self‐consistent iteration was set to be 10^−5^ eV. The atomic positions were fully relaxed until the force on each atom was < 0.02 eV Å^−1^. Dispersion interactions were described using Grimme's DFT‐D3 methodology. The vacuum spacing perpendicular to the plane of the structure is 15 Å. The Perdew (Burke) Ernzerhof generalized gradient approximation (GGA) exchange‐correlation functional was used throughout. A Monkhorst–Pack scheme of the 3×3×1 k‐point grid was used for integration over the Brillouin zone. The IrO_2_(111), MnO_2_(310), and Ir‐doped MnO_2_(310) slab models with periodic boundary conditions were used.

## Conflict of Interest

The authors declare no conflict of interest.

## Supporting information

Supporting Information

## Data Availability

The data that support the findings of this study are available from the corresponding author upon reasonable request.
