# Supplementary material for: High‐Valence‐Manganese Driven Strong Anchoring of Iridium Species for Robust Acidic Water Oxidation
Source: Adv Sci (Weinh). 2023 Jan 22;10(8):2205920. doi: 10.1002/advs.202205920 (PMC10015899; doi:10.1002/advs.202205920)
Supplement: Supplementary file 1 — Supporting Information [file ADVS-10-2205920-s001.pdf]

ADVANCED SCIENCE
Open Access

Supporting Information

for *Adv. Sci.*, DOI 10.1002/advs.202205920

High-Valence-Manganese Driven Strong Anchoring of Iridium Species for Robust Acidic Water Oxidation

Yuxiao Weng, Keyu Wang, Shiyi Li, Yixing Wang, Linfeng Lei, Linzhou Zhuang and Zhi Xu**

Supporting information

High-valence-manganese driven strong anchoring of iridium species for robust acidic water oxidation

Yuxiao Weng, [£] *Keyu Wang,* [£] *Shiyi Li, Yixing Wang, Linfeng Lei, Linzhou Zhuang**,

*Zhi Xu**

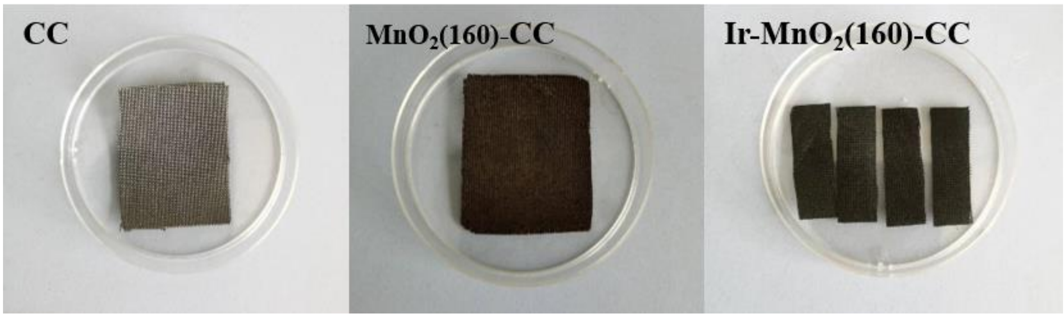

Figure S1. The photos of CC, MnO_2(160)-CC, Ir-MnO_2(160)-CC

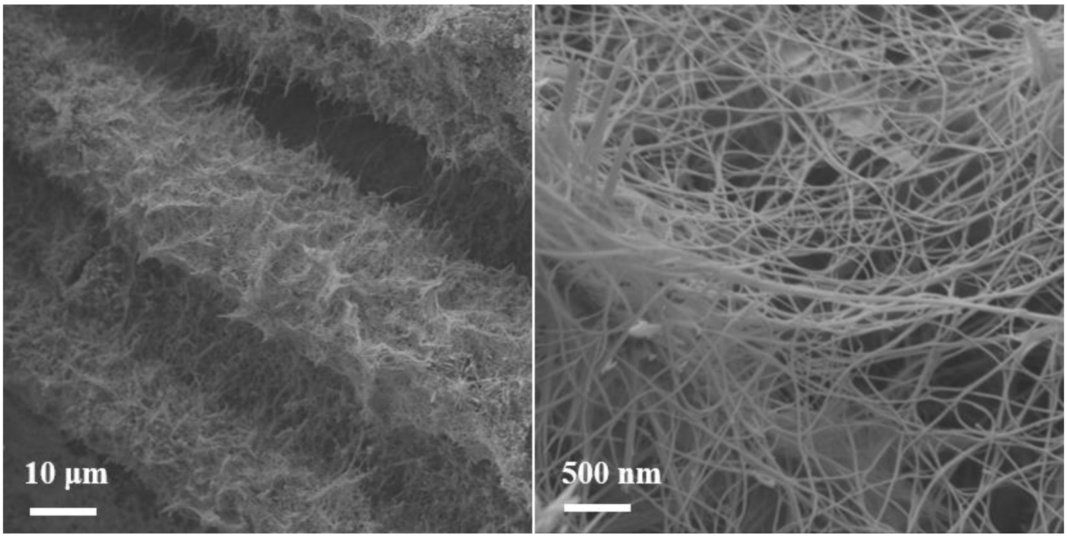

Figure S2. The SEM imagines of Ir-MnO_2(160)-CC.

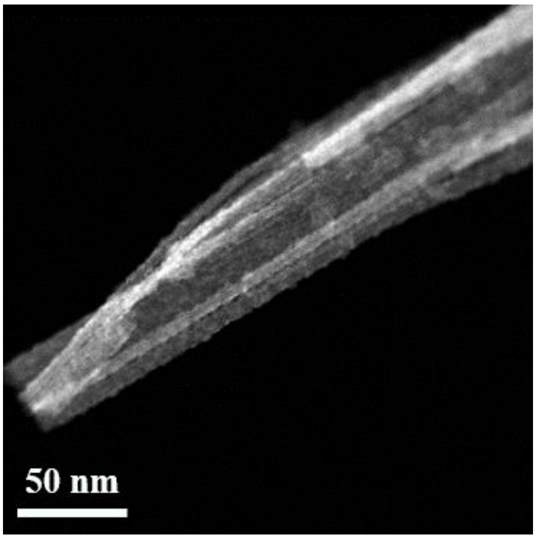

Figure S3. The HAADF STEM of Ir-MnO_2(160)-CC

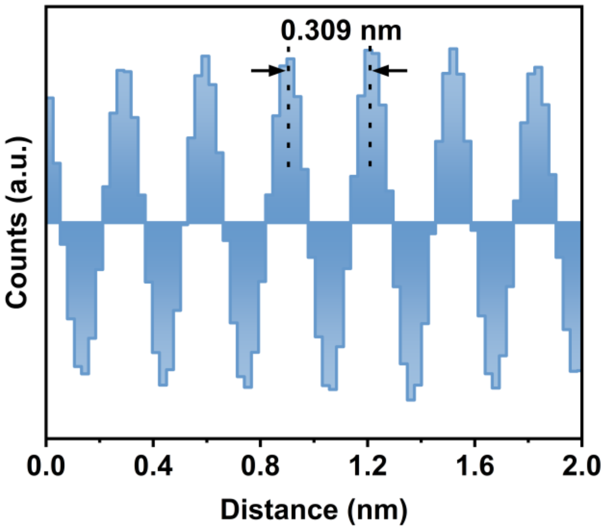

Figure S4. The lattice spacing of MnO_2(160)

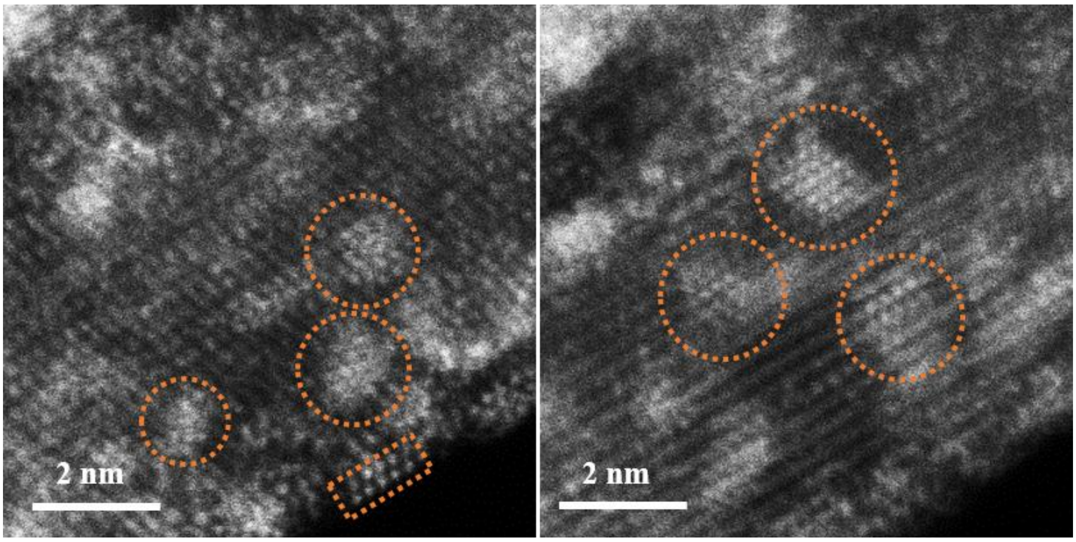

Figure S5. The Ac-TEM of Ir-MnO_2(160)-CC

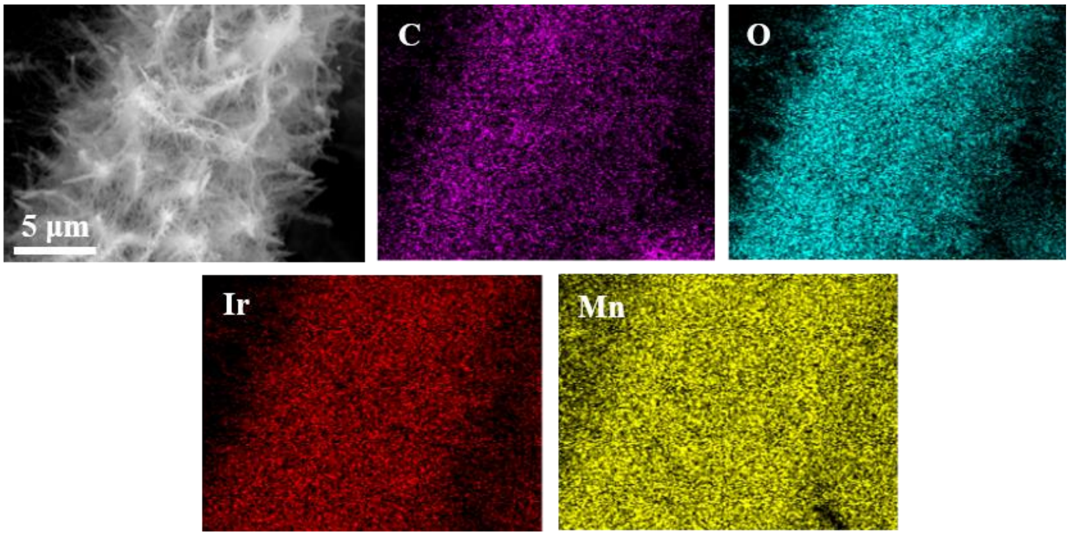

Figure S6. SEM image of Ir-MnO_2(160)-CC and elemental mapping images of C, Ir, and O elements.

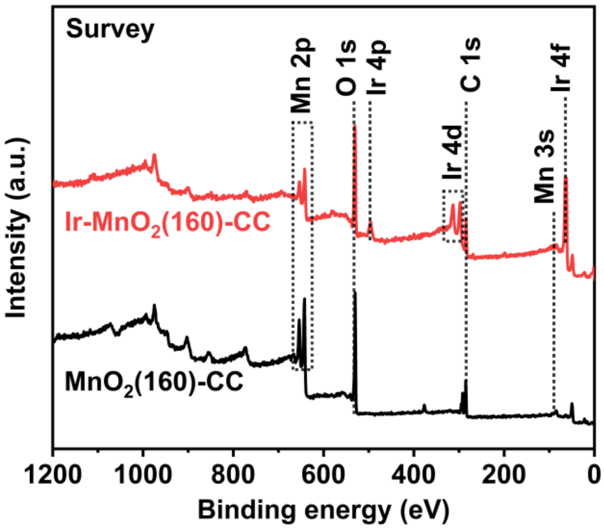

Figure S7. The XPS survey spectra.

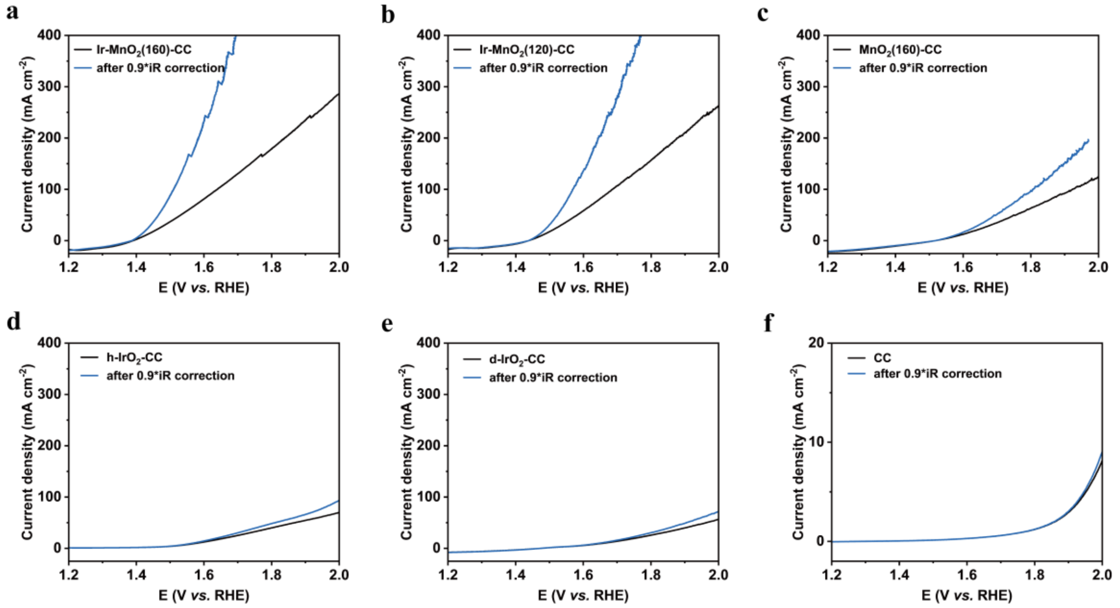

Figure S8. The CV curves of the samples before and after 0.9**iR* correction.

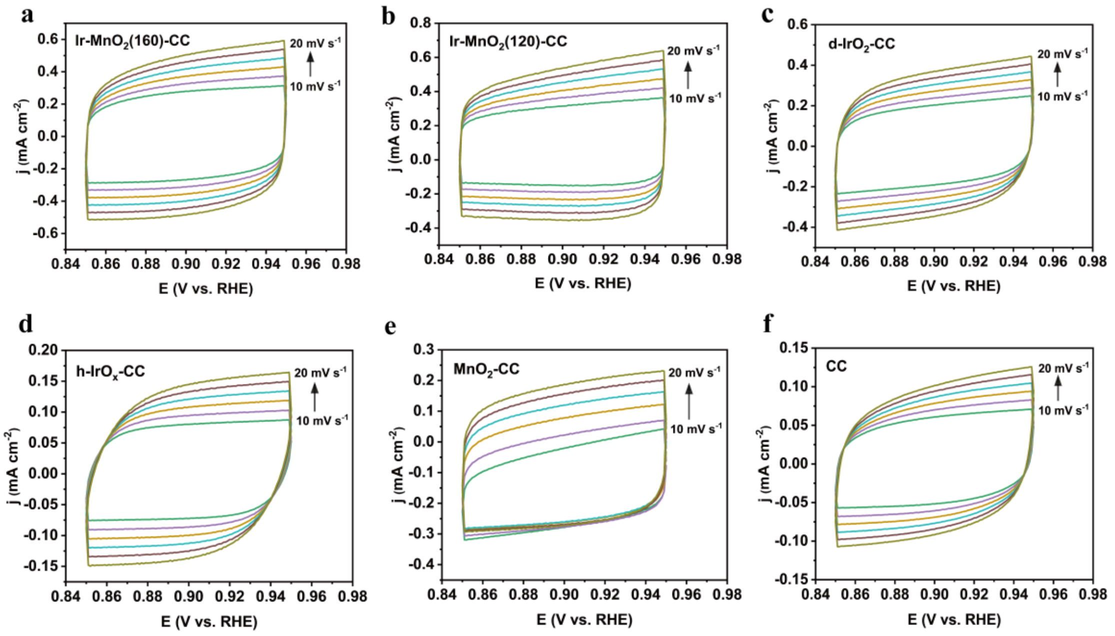

Figure S9. The Cyclic voltammetry curves of catalysts at different scan rates.

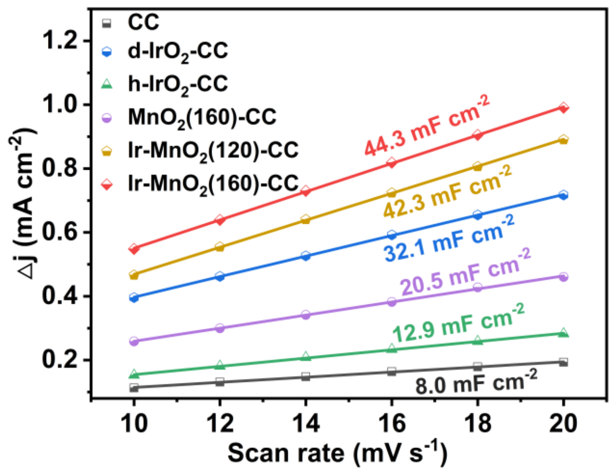

Figure S10. C_{dl} of Ir-MnO_2(160)-CC, Ir-MnO_2(120)-CC, h-IrO_2-CC and d-IrO_2-CC for OER.

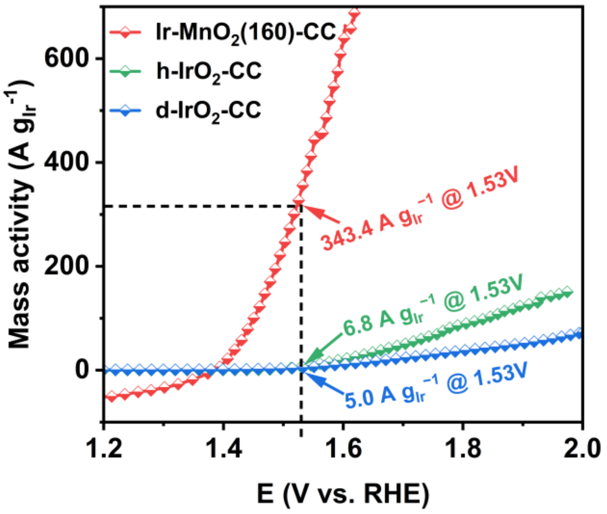

Figure S11. Mass activity of Ir-MnO_2(160)-CC, h-IrO_2-CC and d-IrO_2-CC for OER.

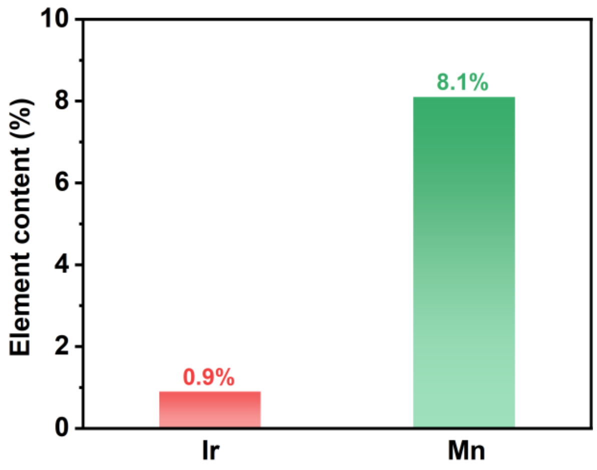

Figure S12. Element contents of Ir and Mn for Ir-MnO_2(120)-CC.

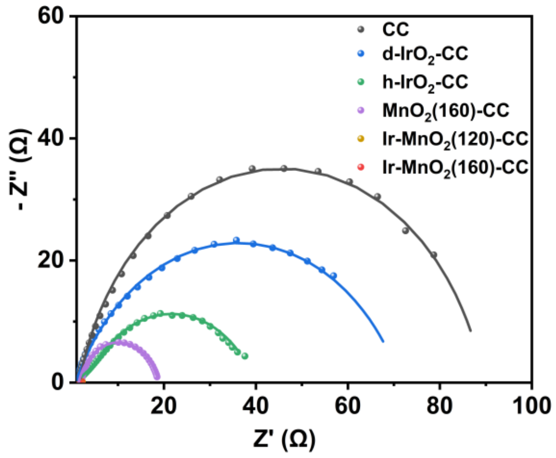

Figure S13. The EIS curve of catalysts.

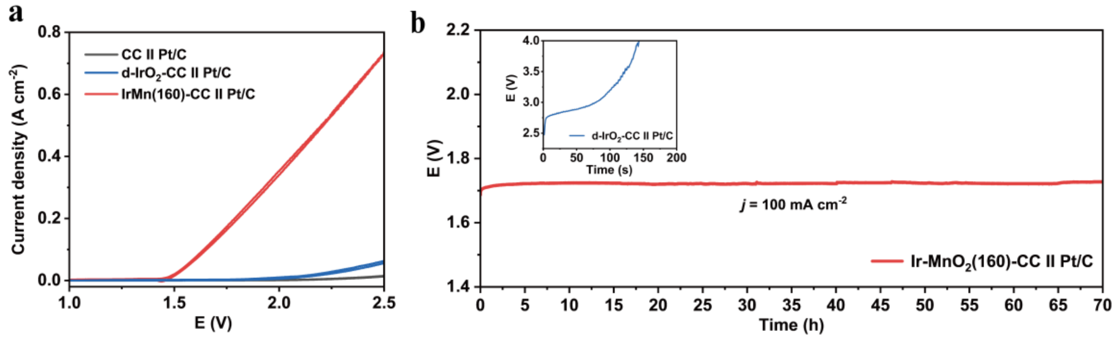

Figure S14. a) The OER performance of catalysts tested in 0.5 M H_2SO_4 solution in PEMWE. b) The stability of catalysts tested in 0.5 M H_2SO_4 solution in PEMWE.

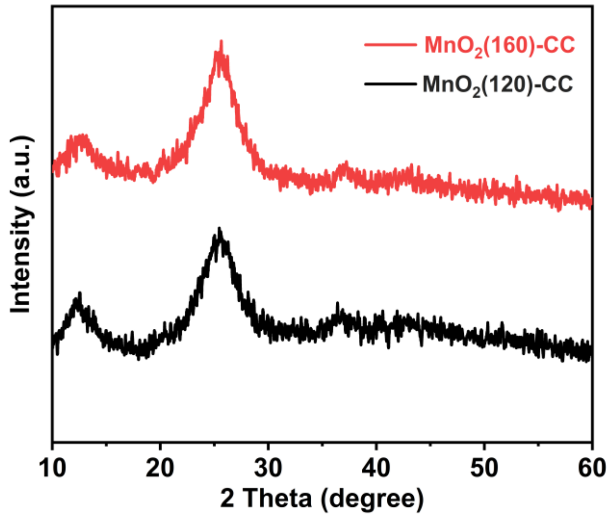

Figure S15. The XRD patterns of MnO_2(160)-CC and MnO_2(120)-CC

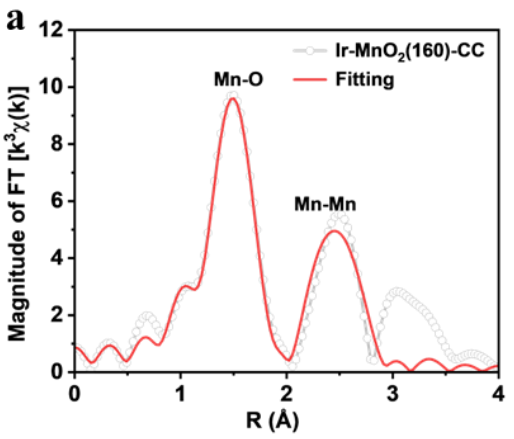

Figure S16. The FT curves of Mn *R*-space of Ir-MnO_2(160)-CC and Ir-MnO_2(120)-CC.

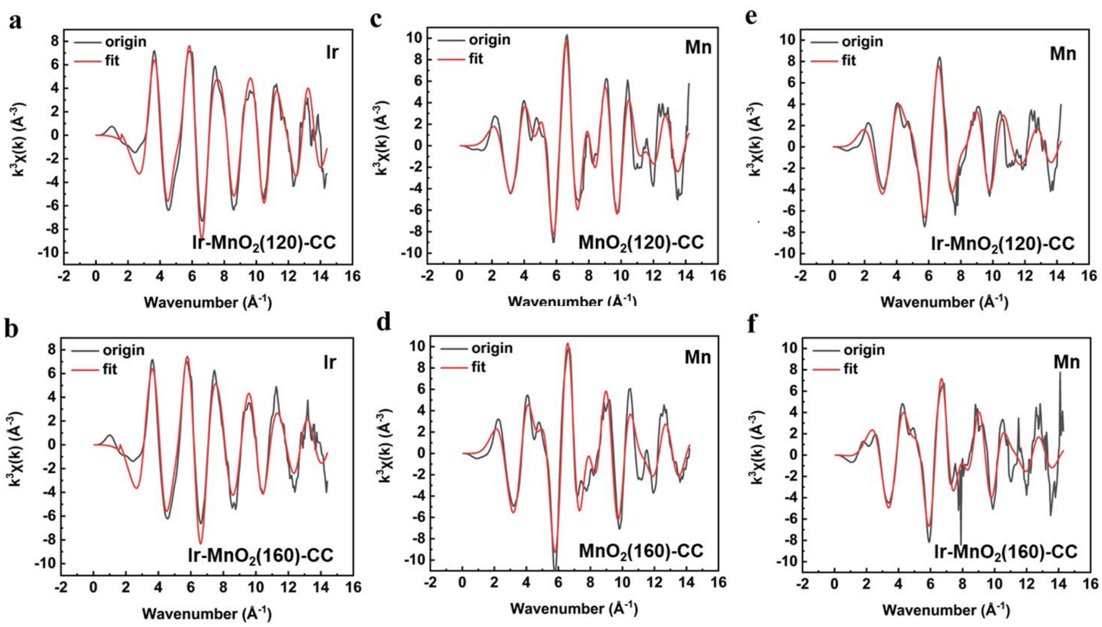

Figure S17. The Ir *k*-space χ(k) curves of EXAFS oscillation functions for Ir-MnO_2(120)-CC(a) and Ir-MnO_2(160)-CC (b), and the Mn *k*-space χ(k) curves of EXAFS oscillation functions for MnO_2(120)-CC(c), MnO_2(160)-CC (d), Ir-MnO_2(120)-CC(e) and Ir-MnO_2(160)-CC(f).

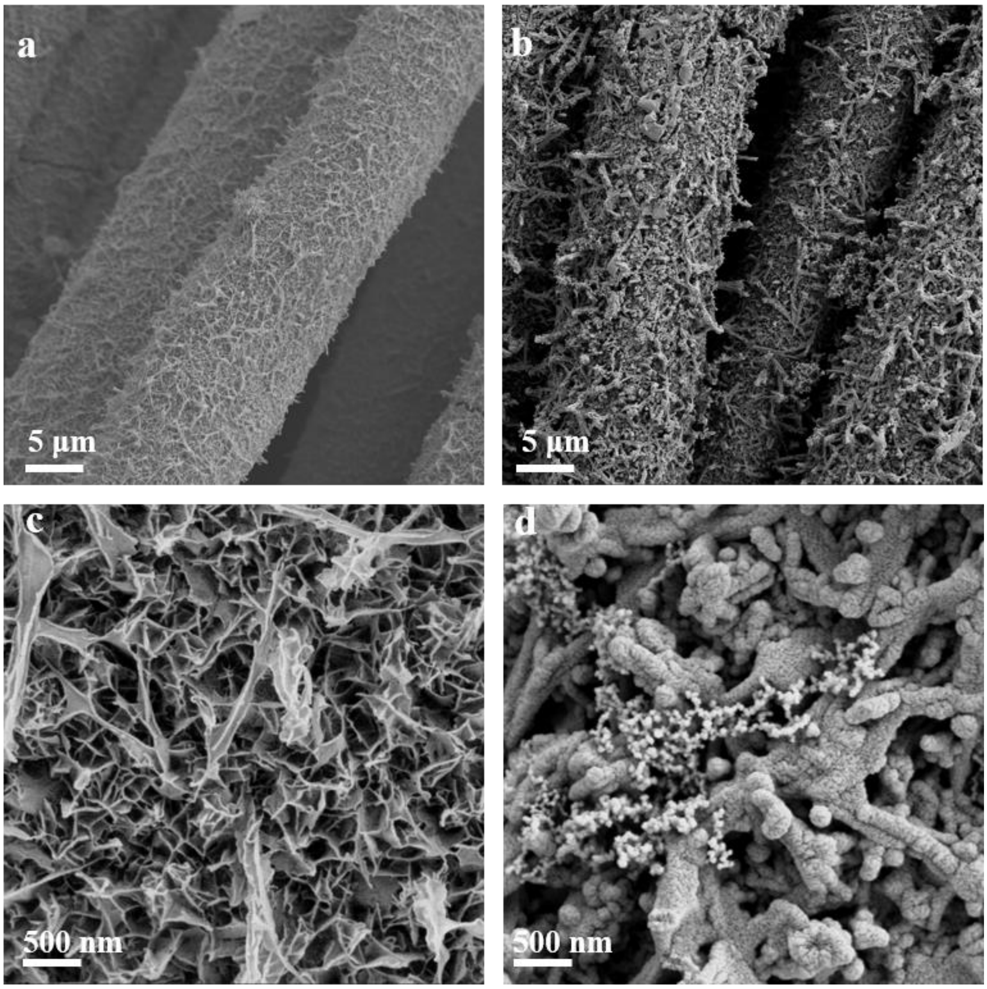

Figure S18. The SEM images. a) and b) MnO_2(120)-CC; c) and d) Ir-MnO_2(120)-CC.

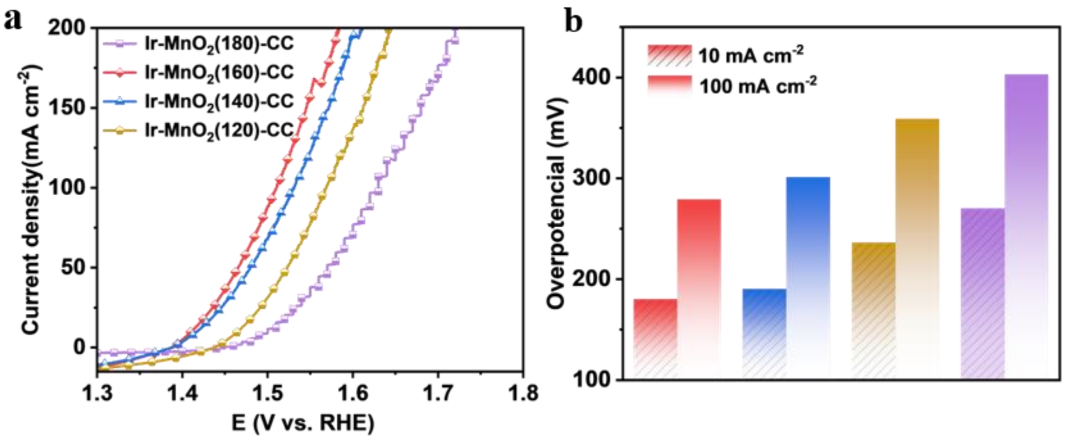

Figure S19. Electrochemcial test for Ir-MnO_2 at different hydrothermal temperature. (a) CV curve obtained at a scan rate of 5 mV s^{-1}, (b) overpotential required to deliver 10 mA cm^{-2} and 100 mA cm^{-2}.

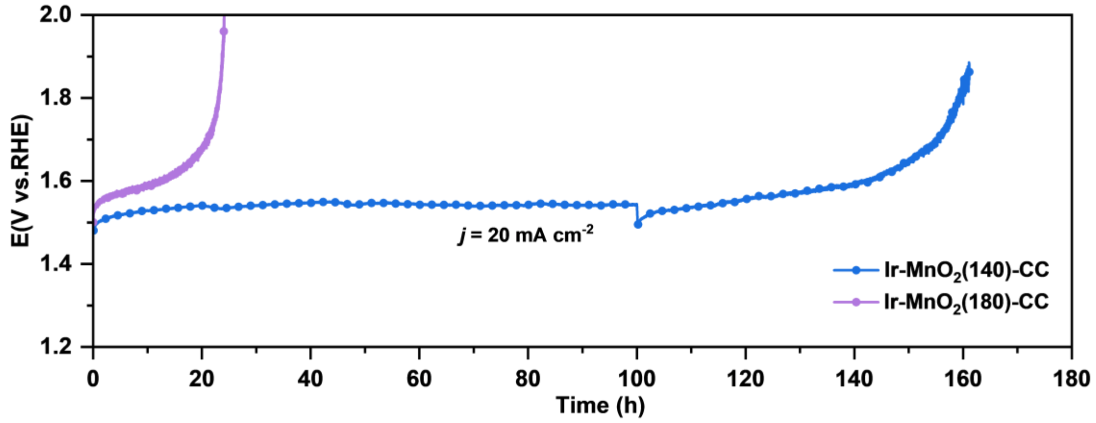

Figure S20. Chronopotentiometric response for OER at 20 mA cm^{-2}.

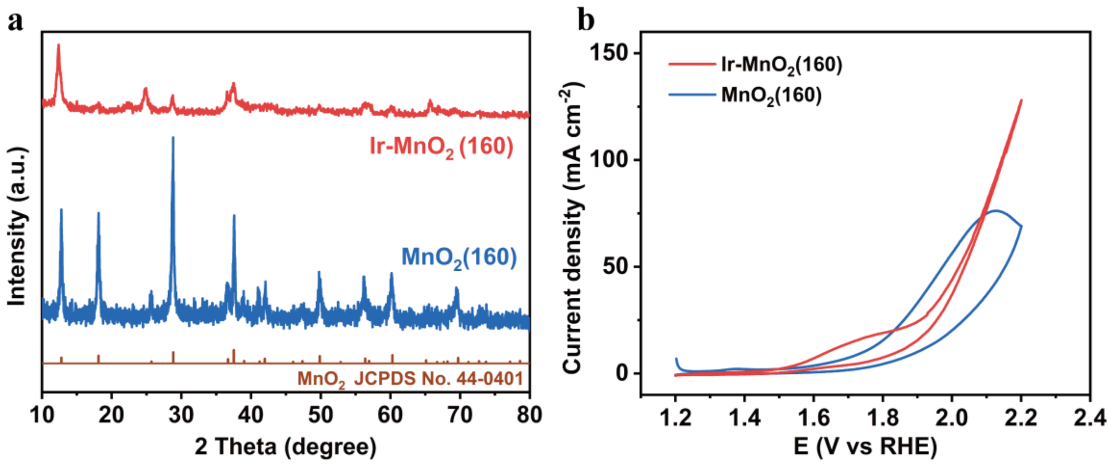

Figure S21. a) the XRD pattern. b) the CV of the MnO_2(160) powder and Ir-MnO_2(160) powder.

Without carbon cloth, the Mn-O-Ir coordination may not be formed following the same procedure. Consequently, in Figure S18, the XRD results show that Ir species

could not be anchored onto MnO_2 powder, and CV showed that the obtained catalysts have much poorer OER performance compared with that supported on carbon cloth.

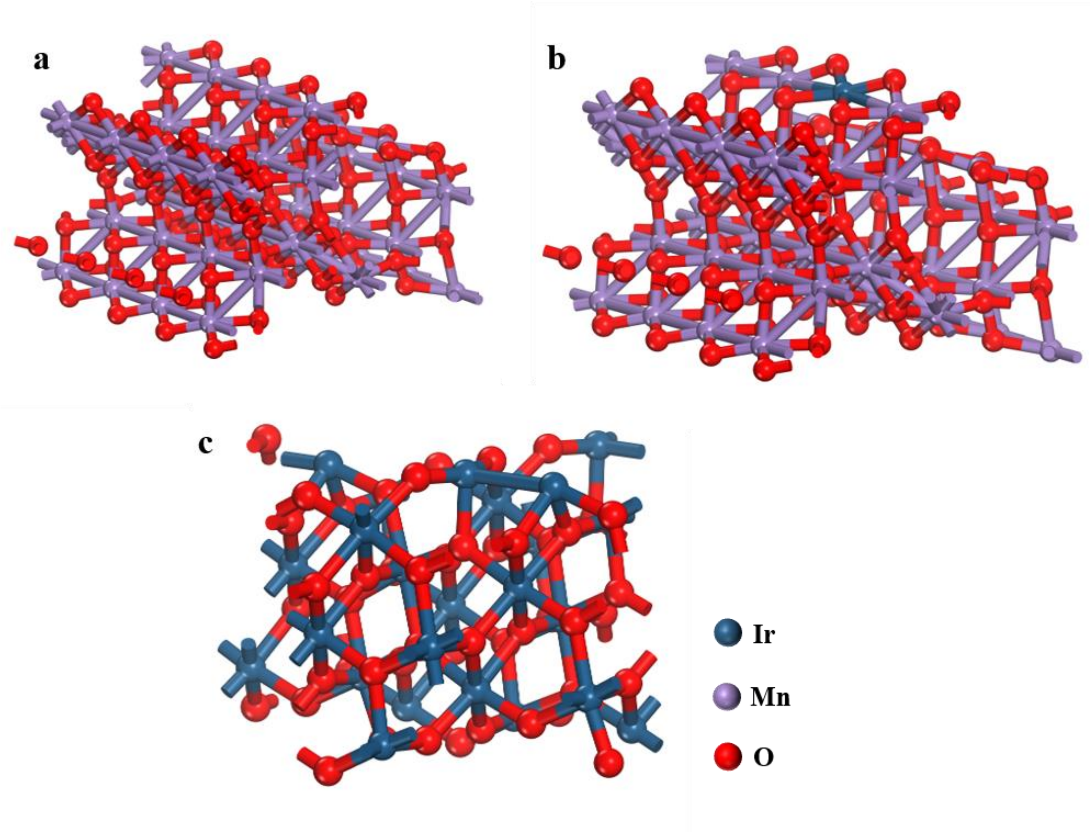

Figure S22. Crystal structure of calculated configurations. a) MnO_2. b) Ir-MnO_2. c) IrO_2.

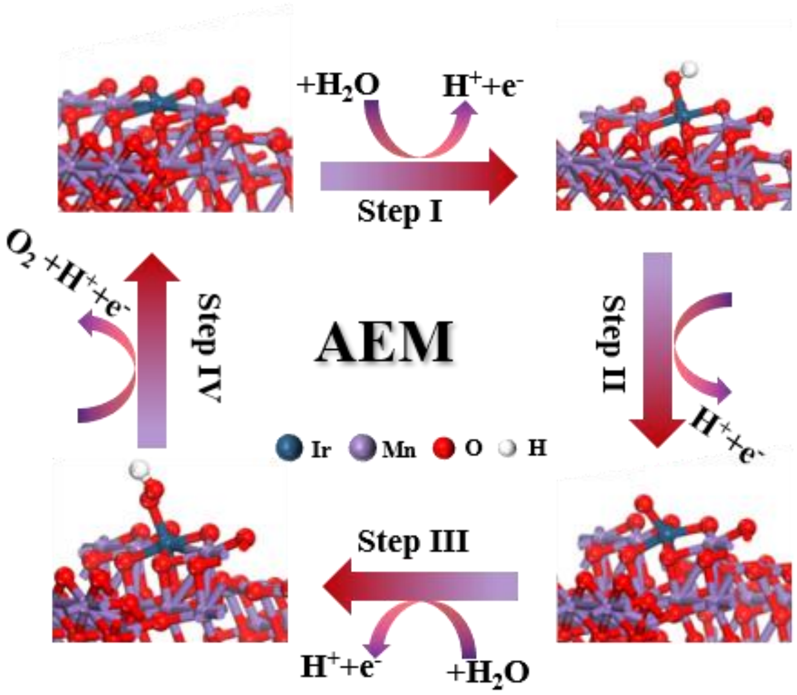

Figure S23. Pathways of AEM.

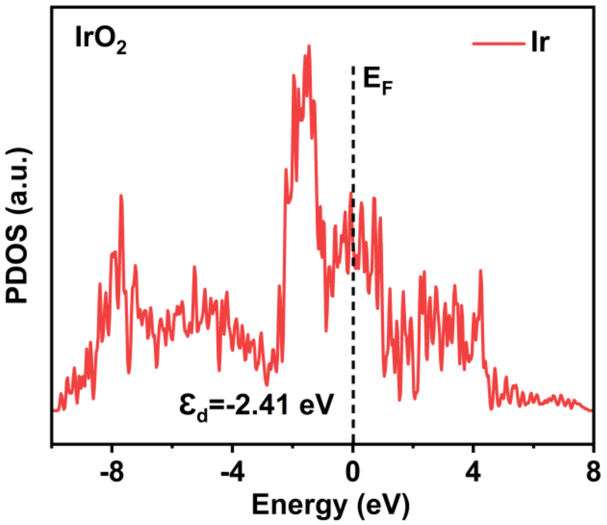

Figure S24. Projected DOS plots of Ir (d) for IrO_2 (111).

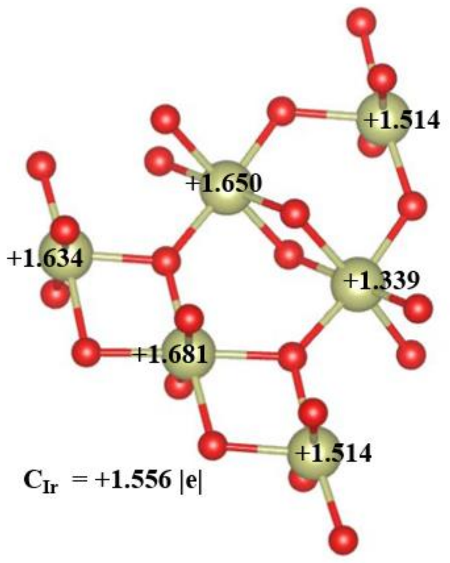

Figure S25. Differential charge density at Ir sites in IrO_2 (111).

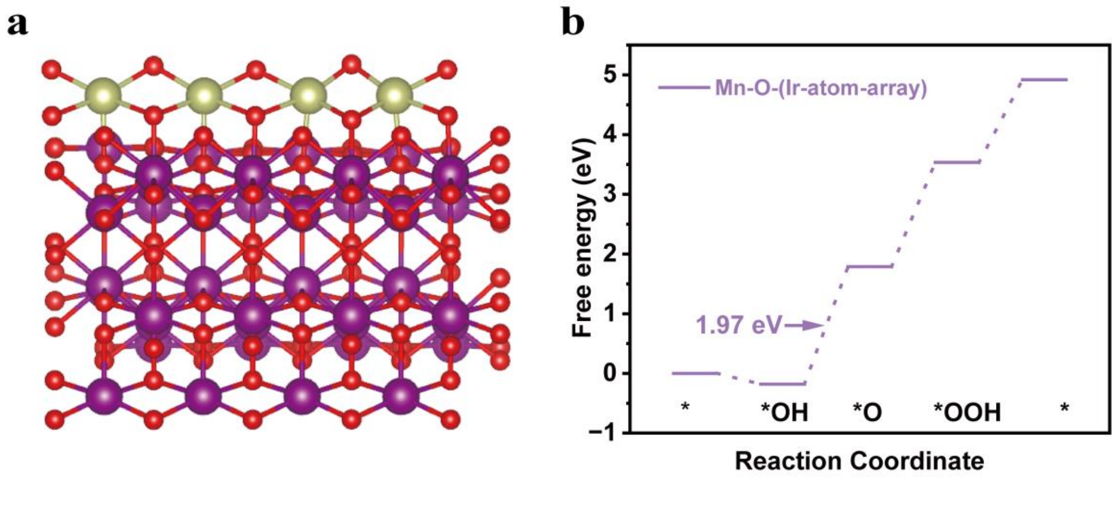

Figure S26. a). Calculated configurations of Mn-O-(Ir-atom-array) structure. b) The Gibbs free-energy diagram.

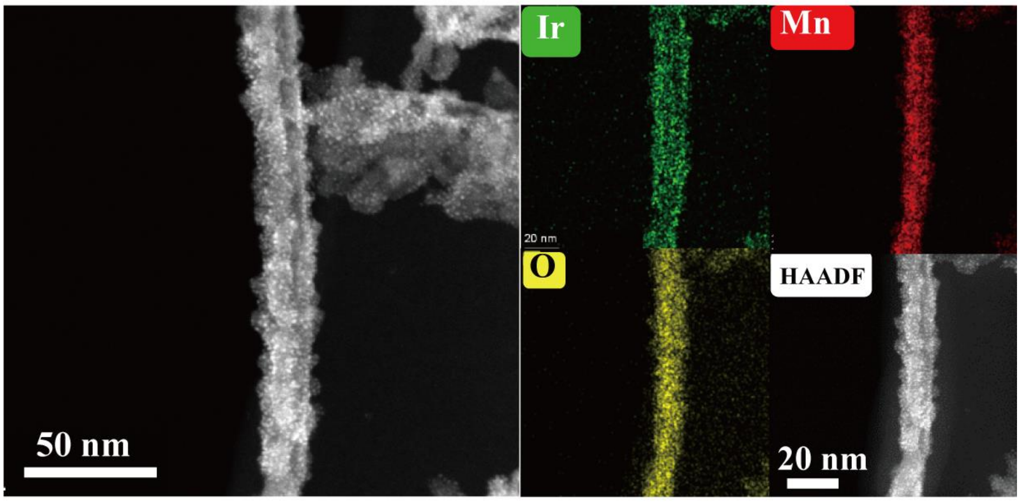

Figure S27. TEM image of spent Ir-MnO_2(160)-CC and corresponding mapping images.

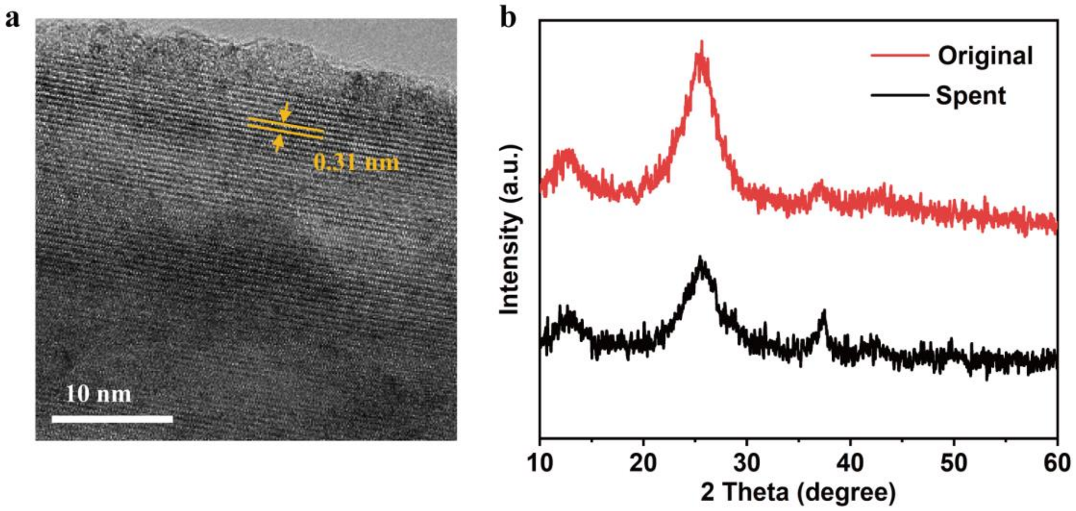

Figure S28. a) The HRTEM of spent Ir-MnO_2(160)-CC. b) XRD pattern for original and spent of Ir-MnO_2(160)-CC.

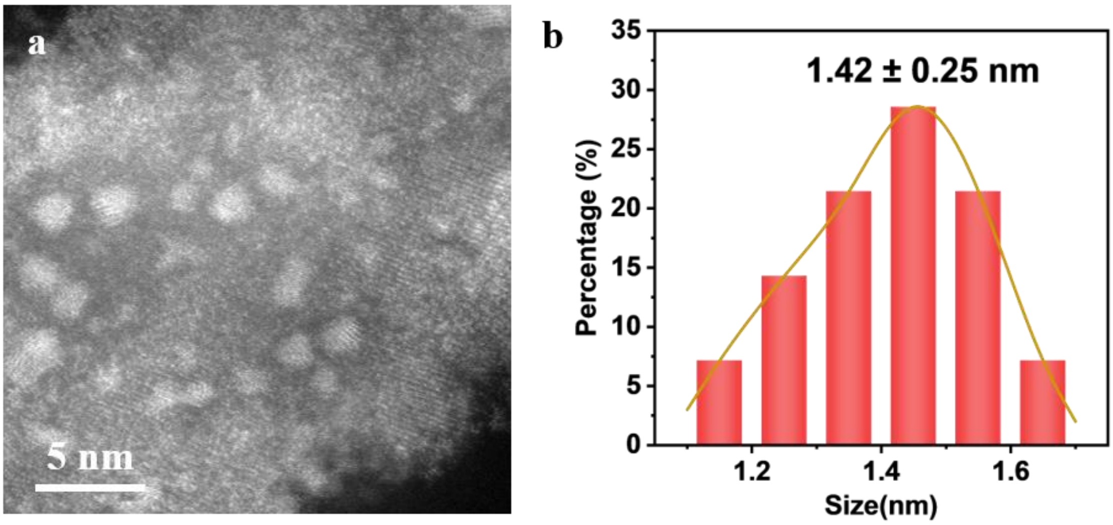

Figure S29. a) Aberration-corrected HAADF-STEM image of spent Ir-MnO_2(160)-CC. b) The size distribution.

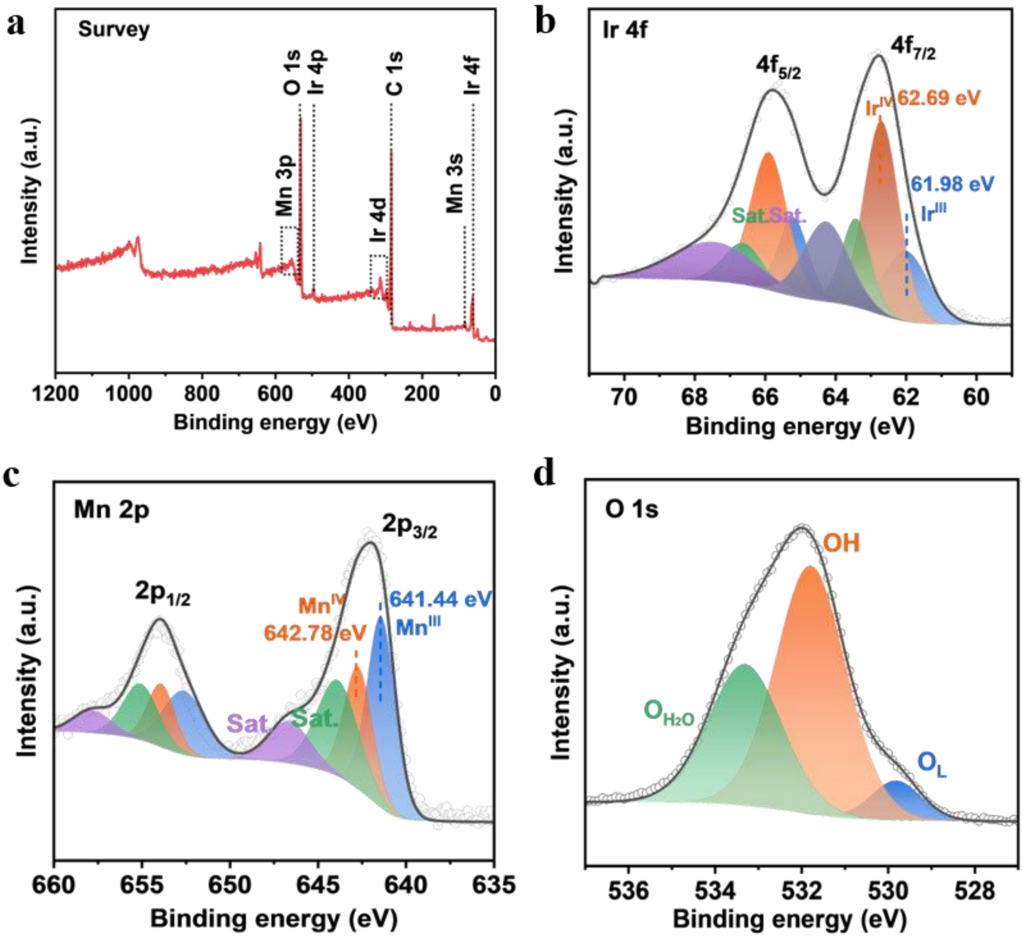

Figure S30. XPS spectra of spent Ir-MnO_2(160)-CC. a) The XPS survey spectra. b) Ir 4f XPS spectra. c) Mn 2p XPS spectra. d) O 1s XPS spectra.

Table S1. The mass concentration of Ir and Mn in Ir-MnO_2-CC with different reaction temperatures by ICP-OES measurement.

Samples	Ir (%)	Mn (%)
Ir-MnO_2(160)-CC	2.7	7.7
Ir-MnO_2(140)-CC	2.3	7.9
Ir-MnO_2(120)-CC	0.9	8.1
Ir-MnO_2(180)-CC	0.4	9.0

Table S2. Comparison of the OER performance compared to previously reported catalysts in acid media.

catalysts	η_{10}(mV)	Tafel slope [mV dec^{-1}]	Stability (h)	Ref.
**Ir-MnO_2(160)-CC**	181	74	180 h @ 20 mA cm^{-2}	This work
**Ir-MnO_2(140)-CC**	190	75.8	120 h @ 20 mA cm^{-2}	This work
**Ir-MnO_2(120)-CC**	236	87.4	20 h @ 20 mA cm^{-2}	This work
**Ir-MnO_2(180)-CC**	270	93.4	15 h @ 20 mA cm^{-2}	This work
**6H-$SrIrO_3$**	248	/	30 h @ 10 mA cm^{-2}	S1
**IrO_xQD/GDY**	236	26	3000 cycles	S2
**Ir-doped WO_3**	258	48	60 h@100 mA cm^{-2}	S3
**IrO_2/V_2O_5**	266	56	20 h@ 10 mA cm^{-2}	S4
**Ir−$NiCo_2O_4$ NSs**	240	60	70 h @ 10 mA cm^{-2}	S5
**$(Mn_{0.8}Ir_{0.2})O_2$ 10F**	220	38	24 h @ 1.45 V (vs RHE)	S6
**Ir/TiO_2-MoO_x**	290	/	10 h @ 5 mA cm^{-2}	S7
**RuO_2/$(Co,Mn)_3O_4$**	270	77	24 h @ 10 mA cm^{-2}	S8
**Mn-RuO_2**	158		5000 cycles	S9
**12Ru/MnO_2**	161	29.4	200 h @ 10 mA cm^{-2}	S10
**$Mn_{0.73}Ru_{0.27}O_{2-\delta}$**	208	65.3	10 h @ 10 mA cm^{-2}	S11
**Amorphous Ir NSs**	255	40	24 h @ 1.45 V	S12

**IrCuNi**	273 mV	41	10 h @ 1.5 V	S13
**$RuIrO_x$**	220	42	24 h @ 1.45 V	S14

Table S3 C_{dl} and ECSA data of catalysts in OER tests.

	C_{dl} (mF cm^{-2})	ECSA (cm_{ECSA}^2)
Ir-MnO_2(160)-CC	44.3	738.3
Ir-MnO_2(120)-CC	42.3	705
MnO_2(160)-CC	20.5	341.7
h-IrO_2-CC	12.94	215.7
d- IrO_2-CC	32.16	536
CC	8.02	133.7

Table S4. The corresponding parameter of the fitting equivalent circuit.

Samples	R_s(Ω)	R_{ct}(Ω)
Ir-MnO_2(160)-CC	1.35	0.85
Ir-MnO_2(120)-CC	1.30	1.07
MnO_2-CC	1.45	14.7
h-IrO_2-CC	1.25	36.1
d-IrO_2-CC	1.30	71.5
CC	1.25	88.7

Table S5. The EXAFS fitting parameters of Ir R-space for Ir-MnO_2(160)-CC and Ir-MnO_2(120)-CC, and Mn R-space for MnO_2(160)-CC and MnO_2(120)-CC.

Catalyst	R-space	Scattering path	CN	R(Å)	σ^2	R-factor
Ir-MnO_2(160)-CC	Ir	Ir-O	5.90	2.0	0.00452	0.006
		Ir-Mn	5.54	2.99	0.01423	
		Ir-Ir	4.49	3.16	0.02014	
	Mn	Mn-O	3.65	1.9	0.00487	0.009
		Mn-Mn	3.61	2.89	0.01057	
Ir-MnO_2(120)-CC	Ir	Ir-O	5.40	1.99	0.00271	0.005
		Ir-Mn	4.42	3.0	0.01026	
		Ir-Ir	6.07	3.12	0.01764	
	Mn	Mn-O	4.37	1.89	0.00144	0.017
		Mn-Mn	4.06	2.86	0.00961	
MnO_2(160)-CC	Mn	Mn-O	4.72	1.90	0.00415	0.009
		Mn-Mn	4.74	2.88	0.00833	
MnO_2(120)-CC	Mn	Mn-O	3.55	1.90	0.00352	0.011
		Mn-Mn	5.22	2.87	0.00546	

References

[S1] L. Yang, G. Yu, X. Ai, W. Yan, H. Duan, W. Chen, X. Li, T. Wang, C. Zhang, X. Huang, J.-S. Chen, X. Zou, *Nat. Commun.* **2018**, 9, 5236.

[S2] Z. Wang, Z. Zheng, Y. Xue, F. He, Y. Li, *Adv. Energy Mater.* **2021**, 11, 2101138.

[S3] P. Li, X. Duan, Y. Kuang, X. Sun, *Small* **2021**, 17, 2102078.

[S4] X. Zheng, M. Qin, S. Ma, Y. Chen, H. Ning, R. Yang, S. Mao, Y. Wang, *Adv. Sci.* **2022**, 9, 2104636.

[S5] J. Yin, J. Jin, M. Lu, B. Huang, H. Zhang, Y. Peng, P. Xi, C.-H. Yan, *J. Am. Chem. Soc.* **2020**, 142, 18378.

[S6] S. D. Ghadge, O. I. Velikokhatnyi, M. K. Datta, P. M. Shanthi, S. Tan, K. Damodaran, P. N. Kumta, *ACS Catal.* **2019**, 9, 2134.

[S7] E.-J. Kim, J. Shin, J. Bak, S. J. Lee, K. h. Kim, D. Song, J. Roh, Y. Lee, H. Kim, K.-S. Lee, E. Cho, *Appl. Catal. B* **2021**, 280, 119433.

[S8] J. He, W. Li, P. Xu, J. Sun, *Appl. Catal. B* **2021**, 298, 120528.

[S9] S. Chen, H. Huang, P. Jiang, K. Yang, J. Diao, S. Gong, S. Liu, M. Huang, H. Wang, Q. Chen, *ACS Catal.* **2020**, 10, 1152.

[S10]C. Lin, J.-L. Li, X. Li, S. Yang, W. Luo, Y. Zhang, S.-H. Kim, D.-H. Kim, S. S. Shinde, Y.-F. Li, Z.-P. Liu, Z. Jiang, J.-H. Lee, *Nat. Catal.* **2021**, 4, 1012.

[S11]K. Wang, Y. Wang, B. Yang, Z. Li, X. Qin, Q. Zhang, L. Lei, M. Qiu, G. Wu, Y. Hou, *Energy Environ. Sci.* **2022**, 15, 2356.

[S12]G. Wu, X. Zheng, P. Cui, H. Jiang, X. Wang, Y. Qu, W. Chen, Y. Lin, H. Li, X. Han, Y. Hu, P. Liu, Q. Zhang, J. Ge, Y. Yao, R. Sun, Y. Wu, L. Gu, X. Hong, Y. Li, *Nat. Commun.* **2019**, 10, 4855.

[S13] D. Liu, Q. Lv, S. Lu, J. Fang, Y. Zhang, X. Wang, Y. Xue, W. Zhu, Z. Zhuang, *Nano Lett.* **2021**, 21, 2809.

[S14] Z. Zhuang, Y. Wang, C.-Q. Xu, S. Liu, C. Chen, Q. Peng, Z. Zhuang, H. Xiao, Y. Pan, S. Lu, R. Yu, W.-C. Cheong, X. Cao, K. Wu, K. Sun, Y. Wang, D. Wang, J. Li, Y. Li, *Nat. Commun.* **2019**, 10, 4875.
